# Stressed Yeast Paint a Picture of Dorian Gray

**DOI:** 10.1371/journal.pbio.1001885

**Published:** 2014-06-17

**Authors:** Roland G. Roberts

**Affiliations:** Public Library of Science, Cambridge, United Kingdom

**Figure:**
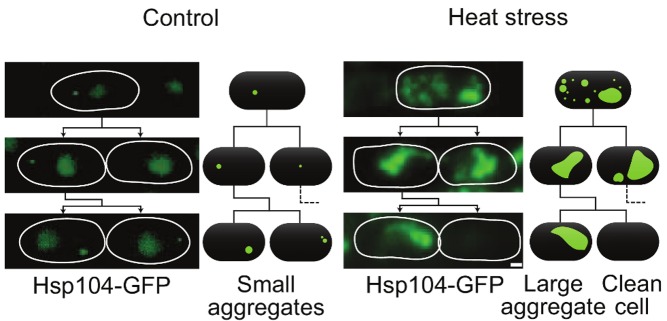
Stress breeds asymmetry. In the normal situation (left-hand panel), aggregates are small and partitioned symmetrically. After thermal stress (right-hand panel), increased rates of aggregate fusion result in a smaller number of larger aggregates. By the second cell division (bottom of each panel), one cell inherits all, and is likely to die soon after.

We all pass unwanted stuff on to our children—emotional baggage, peculiar habits, unfashionable furniture. Cells do the same thing when they divide; along with their newly replicated genomes and the vital cellular organelles, they also pass on a lifetime's worth of accrued rubbish. An important component of this is the legacy of insoluble aggregated protein that the parent cell has failed to deal with by the normal processes of degradation, and the amount that's passed on can affect the lifespan and wellbeing of the daughter cells.

In the case of cells that divide asymmetrically, such as baker's yeast (*Saccharomyces cerevisiae*), the mother cell selflessly keeps the junk to herself, leaving her daughters pristine. This is known to happen by an active process of aggregate retention. But what about symmetrically dividing cells? Who gets mum's trash there?

The fission yeast *Schizosaccharomyces pombe* normally divides symmetrically, even when it comes to protein aggregates. In general the dilution that happens at cell division means that the progeny can cope with this burden, but it's been shown that when protein aggregation is dramatically increased by a stressful event, such as heat shock (think about the protein aggregation that occurs when you poach an egg), *S. pombe* switches to dividing up its aggregates *asymmetrically*. How does it do this?

A new study by Miguel Coelho, Iva Tolić, and colleagues, just published in *PLOS Biology*, reveals that the yeast has found a surprisingly simple and elegant solution to the problem of waste management. The authors study what happens to protein aggregates in *S. pombe*, following their fate by using a fluorescently tagged version of the disaggregase enzyme Hsp104, which is known to associate with aggregated proteins.

First the authors look at the basic behaviour of aggregates—they find that these seem to drift around the cell by diffusion alone, that the aggregates rarely split up, and that almost every encounter between two aggregates results in their fusion to form a single larger one. When the cells divide symmetrically, the aggregates are on average distributed equally between the daughters. And when asymmetry is forced by using a mutant yeast strain (Δ*pom1*) which divides off-centre, then the asymmetry of the aggregate inheritance tracks the asymmetry of the cell volumes.

These data are consistent with the movement and inheritance of protein aggregates being a simple stochastic process. The authors model this mathematically, assuming Brownian motion of the aggregate particles and the existence of ‘invisible’ aggregates, too small to be seen by the authors' imaging system, which progressively fuse with others until they pass a visibility threshold. They find that the model closely reproduces the properties of real aggregates in real cells, suggesting that no further mechanisms are needed to explain their behaviour.

But what happens when the cells are stressed? The authors subject the yeast to two types of stress: oxidative stress (exposure to hydrogen peroxide) or heat shock (a short period at 40°C). In each case, the number of aggregates increases, but crucially, and as predicted by the model, the number of fusion events also increases substantially. So much so, in fact, that within a couple of cell divisions, all of the aggregates end up in a single large clump. This clump is inherited by one of the daughter cells, which usually then dies, leaving the other daughter aggregate-free and ready to start a new life.

The authors then try to identify what's responsible for the fusion process. They light upon Hsp16, a small heat-shock protein known to associate with aggregated proteins during times of stress. They find that aggregates in cells lacking the Hsp16 gene undergo fewer fusion events overall, with only half of the encounters between aggregates resulting in fusion. This connection between Hsp16 and aggregate fusion seems highly specific—fission and nucleation aren't affected by Hsp16 loss, and deletion of two other heat-shock genes doesn't affect fusion. Without Hsp16, fusion is less efficient, the number of aggregates per cell is higher after stress, partition of aggregates at cell division is more symmetrical, half as many cells are born without aggregates, and more cells die overall.

The overall picture painted by the cellular experiments and the mathematical modelling is surprisingly simple. During normal conditions, protein aggregates drift around, bump into each other and fuse occasionally, are shared more-or-less equally between daughter cells, and are sufficiently diluted by cell division not to interfere with a healthy existence. After stress, however, when more aggregates are created, the likelihood of two aggregates meeting and fusing increases, giving rise to ever larger clumps. After one or two cell divisions, they all end up in one massive cluster, and this can only pass to one of the daughter cells; one cell is left as fresh and sparkling as a newborn baby, and the other takes the full hit of a history of aggregation.

Thus when times get tough, simple diffusion, chaperoned fusion of particles and the vicissitudes of stochasticity can give rise to an adaptively valuable switch from symmetric division—two gracefully aging twins—to asymmetric division—the eternally young and beautiful Dorian Gray, and his corrupt and damaged portrait in the attic.

**Coelho M, Lade SJ, Alberti S, Gross T, Tolić IM (2014) Fusion of Protein Aggregates Facilitates Asymmetric Damage Segregation.** doi:10.1371/journal.pbio.1001886

